# Determinants of E2-ubiquitin conjugate recognition by RBR E3 ligases

**DOI:** 10.1038/s41598-017-18513-5

**Published:** 2018-01-08

**Authors:** Luigi Martino, Nicholas R. Brown, Laura Masino, Diego Esposito, Katrin Rittinger

**Affiliations:** 1 The Francis Crick Institute, 1 Midland Road, London, NW1 1AT UK; 2 Structural Biology Science Technology Platform, The Francis Crick Institute, 1 Midland Road, London, NW1 1AT UK; 3 Present Address: School of Cellular and Molecular Medicine, University of Bristol, University Walk, Bristol, BS8 1TD UK

## Abstract

RING-between-RING (RBR) ubiquitin ligases work with multiple E2 enzymes and function through an E3-ubiquitin thioester intermediate. The RBR module comprises three domains, RING1, IBR and RING2 that collaborate to transfer ubiquitin from the E2~Ub conjugate, recognised by RING1, onto a catalytic cysteine in RING2 and finally onto the substrate in a multi-step reaction. Recent studies have shown that RING1 domains bind E2~Ub conjugates in an open conformation to supress ubiquitin transfer onto lysine residues and promote formation of the E3 thioester intermediate. However, how the nature of the E2 influences the ubiquitin transfer process is currently unclear. We report here a detailed characterization of the RBR/E2-conjugate recognition step that indicates that this mechanism depends on the nature of the E2 enzyme and differs between UbcH5 and UbcH7. In the case of UbcH5~Ub an interaction with ubiquitin is necessary to stabilize the transfer complex while recognition of UbcH7~Ub is driven primarily by E2-RING1 contacts. Furthermore our analysis suggests that RBRs, in isolation and in complex with ubiquitin-loaded E2s, are dynamic species and that their intrinsic flexibility might be a key aspect of their catalytic mechanism.

## Introduction

**Figure 1:**
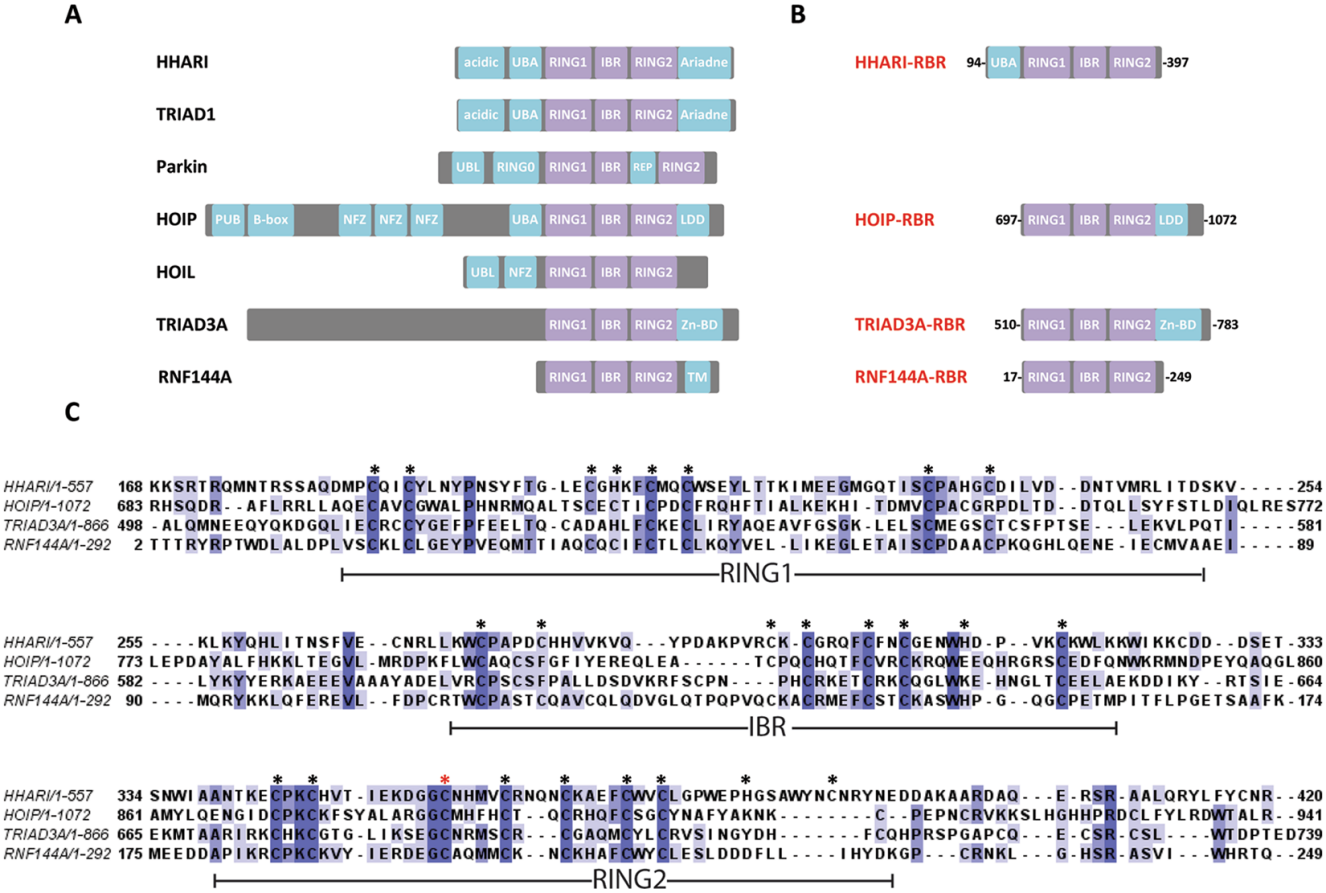
Domain organisation and sequence alignment of RBRs. (**A**) Schematic representation of the domain organisation of some RBR E3 ubiquitin ligases. The RBR domains, comprising a RING1, IBR and a RING2 domain are coloured in purple while extra domains are indicated in cyan. (**B**) The RBR constructs used in this study are reported; for some extra domains had to be included in the recombinant proteins to improve solubility/stability or to preserve enzymatic activity. (**C**) Primary sequence alignment of RBR domains of human HHARI (Uniprot Q9Y4X5), HOIP (Q96EP0), TRIAD3A (Q9NWF9) and RNF144A (P50876) performed by using ClustalOWS and displayed by using JalView program (www.jalview.org). Black stars indicate the residues that are involved in Zinc coordination. The catalytic cysteine residues (C357 for HHARI, C885 for HOIP, C688 for TRIAD3A and C198 for RNF144A) are indicated by a red star.

Attachment of ubiquitin to a protein target is a crucial post-translational modification that regulates a plethora of cellular signalling pathways. It requires the sequential action of three enzymes: ubiquitin activating enzymes (E1), ubiquitin conjugating enzymes (E2) and ubiquitin ligases (E3)^1^. E3s confer substrate specificity and catalyse attachment of ubiquitin onto a lysine residue of the target protein (mono-ubiquitination or chain initiation), or onto a lysine or the N-terminal methionine of another ubiquitin molecule to form polyubiquitin chains. E3s can be classified into two major groups: RING ligases that transfer ubiquitin directly from the E2 conjugate to the target molecule and the thioester-forming E3s, HECT and RBR ligases that function through an E3~ubiquitin (~indicates a thioester bond) thioester intermediate^2–9^. RBR ligases represent the newest addition to the E3 ligase family and their mechanism of action incorporates features of RING and HECT ligases^8^. The catalytic RBR module comprises three zinc-binding motifs: a RING1 domain (the E2-interacting portion) followed by an IBR domain and a RING2 domain that contains the catalytic cysteine (Fig. 1A,B). While the RBR module is well conserved (Fig. 1C), most RBR E3s contain accessory domains N- or C-terminal to the RBR domain, thereby increasing protein complexity and allowing for regulation of enzymatic activity, for example through auto-inhibitory interactions as observed in HHARI and Parkin^10,11^. For other RBRs regulation of catalytic activity is not necessarily achieved through auto-inhibition. For example, even though HOIP in isolation is auto-inhibited by its UBA domain, which is relieved by interaction with HOIL and SHARPIN components of the LUBAC machinery^12–14^, it is now well established that complex formation between LUBAC components is constitutive^15–18^. Instead, LUBAC activity is kept in check by the presence of DUBs, such as OTULIN and CYLD, bound directly (OTULIN) or indirectly (CYLD via SPATA2 protein) to the PUB domain of HOIP^19–30^.

RBR ligases function with multiple E2s, including UbcH5 (UBE2D) isoforms and UbcH7 (UBE2L3), the latter of which has been suggested to be the physiologically relevant E2 for LUBAC and possibly other RBRs^5,8,31–33^. These E2s are mechanistically distinct and while UbcH5 can transfer ubiquitin onto both, lysine and cysteine residues, UbcH7 is strictly cysteine-reactive and hence only functions with RBR and HECT ligases, though it is capable to form stable complexes with RING domains^8,34^. In isolation, UbcH5~Ub exists in multiple conformations but preferentially populates open states^35^. However, only the closed conformation is competent for ubiquitin transfer onto a lysine, which requires interaction with canonical RING domains for its stabilisation^36–39^. In contrast, an open E2~Ub state is stabilized in conjunction with RING1 domains found in RBR E3s^40^. This mode of interaction suppresses lysine reactivity and hence favours transthiolation to form the E3~ubiquitin intermediate. Unlike UbcH5, the UbcH7~Ub conjugate populates closed states to a considerable extent in isolation. However, despite its lack of lysine reactivity, UbcH7~Ub also binds RING1 domains in an open conformation as recently shown for HHARI^40–42^.

Although biochemical studies have advanced our understanding of the mechanistic features of ubiquitin transfer by RBR ligases considerably, structural information on active RBR/E2~Ub complexes is sparse and limited to the crystal structure of the HOIP RBR/UbcH5B-Ub complex (-indicates a stable thioester mimetic, an isopeptide, used for crystallization and biophysical experiments)^43^. This structure (PDB 5EDV) unveiled novel insights into the molecular details of the E2/E3 interaction and provided a model for ubiquitin transfer from E2 to the catalytic cysteine in RING2. Interestingly, the crystal structure of this complex contains two molecules of HOIP-RBR in the asymmetric unit arranged in a domain swapped fashion where each RBR domain binds one molecule of UbcH5B-Ub, plus a free ubiquitin molecule that has been suggested to play an allosteric role (see Supplementary Figure S1A–B). Each RBR molecule adopts an extended conformation (see Supplementary Figure S1C), with RING1 recognising E2 via a surface that is similar, but not identical to the canonical E2/RING interface. UbcH5B-Ub is bound in an open conformation with the ubiquitin contacting the two helices connecting RING1 and IBR. Additional RBR/E2-Ub contacts are made by the IBR-RING2 linker and RING2 from the adjacent complex. However, this intricate arrangement is believed to be a crystallisation artefact, and instead it was suggested that the catalytically relevant unit is formed by half of each of the N- and C-terminal portions of the two RBR molecules bound (see Supplementary Figure S1D, in here referred to as the “compact conformation”). In addition to this structure of an active RBR/E2-Ub complex, two crystal structures of HHARI in complex with UbcH7-Ub were recently described (PDB 5UDH and 5TTE)^41,42^. Although bound to an E2-Ub thioester mimetic, HHARI adopts an autoinhibited conformation in these structures and the catalytic cysteine in RING2 remains occluded by the Ariadne domain. The two structures were solved in different crystal forms and although the overall architecture of the complex is very similar, they differ in the position of the ubiquitin molecule. In both structures UbcH7-Ub interacts with HHARI in an open conformation but in one structure (PDB 5TTE) ubiquitin contacts the UBA domain of the same HHARI molecule in a non-canonical manner, whereas in the other complex (PDB 5UDH), ubiquitin contacts the UBA domain of a symmetry related molecule using an interface that resembles a canonical UBA-ubiquitin interaction^41,42^. Although these structures shed light on E2~Ub recognition by RING1, the presence of the Ariadne domain keeps RING2 away from the E2~Ub conjugate and hence does not provide insight into the active RBR/E2~Ub conformation during the transthiolation step.

The recently described Parkin/phosphoUb complex structures present a partially active form, in which minor conformational changes in the E3 are responsible for the creation of multiple ubiquitin binding sites that may accommodate E2~Ub conjugates^44,45^. Based on these structures a model was proposed, in which Parkin catalyses ubiquitin transfer in *trans* and where an E2~Ub conjugate could bridge two molecules of E3 in a fashion that resembles the arrangement observed in the HOIP/UbcH5B-Ub structure^45^.

In light of these novel structural insights into RBR/E2~Ub complexes and the observation that RING1 domains bind their E2~Ub conjugates in an open conformation in contrast to canonical RING domains that stabilise a closed form, we asked if there are fundamental differences in the way RBRs recognise lysine-reactive UbcH5 enzymes versus cysteine-reactive UbcH7 and how any such differences might influence their catalytic activity. To address these questions we performed an extensive biochemical and biophysical analysis of the RBR domains of HHARI, HOIP, TRIAD3A and RNF144A, in the absence of any potential auto-inhibitory interactions. We characterised their interaction with UbcH7 and UbcH5A, which unveiled what may be a more general feature of RBRs: they all form a strong complex with UbcH7 that is not significantly strengthened upon ubiquitin conjugation. In contrast, no interaction can be detected with isolated UbcH5 and even ubiquitin conjugation only leads to a stable complex for a subset of RBRs. However, these differences in complex formation do not lead to obvious differences in catalytic activity, which may indicate that the only element driving interaction with ubiquitin-loaded E2s is the need for suppression of lysine reactivity of E2s other than UbcH7. Moreover, to provide novel structural insight in the RBR/E2-Ub complexes investigated, we performed an extensive SAXS analysis, which revealed that the RBRs and their complexes with UbcH7-Ub and/or UbcH5A-Ub are monomeric in solution and are highly flexible entities.

## Results

### The RBR domains of HHARI, HOIP, TRIAD3A and RNF144A

For this study, the RBR domains of HHARI, HOIP, TRIAD3A and RNF144A were chosen as representatives of this E3 family (Fig. 1B). For HHARI-RBR, the UBA domain N-terminal to RING1 was included to improve its solubility^10^ while for HOIP-RBR, the C-terminal LDD domain that is required for catalytic activity was retained^12–14^. Less is known about TRIAD3A and RNF144A and sequence alignments were used to define RBR domain boundaries (Fig. 1C). For RNF144A the RBR domain is predicted to span the first 250 residues of the protein, with a trans-membrane element (TM) in the C-terminus of the protein. The 250 residue construct lacking the trans-membrane element was soluble and active and used for this study. The RBR domain of TRIAD3A, predicted to span residues 510 to 714, did not yield a soluble protein; but extending the construct to residue 801 improved solubility. Interestingly, the primary sequence of this C-terminal extension is rich in Cys and His residues and we wondered if they may form an additional zinc binding site. To test this hypothesis, native mass spectroscopy was carried out on a construct encompassing the RING2 of TRIAD3A plus the C-terminal extension (residues 674–801). This analysis indicated the presence of three zinc ions (see Supplementary Figure S2A,B): two of them likely associated with RING2 and a third zinc ion probably bound to the C-terminal extension. We also tested a slightly shorter construct trimmed at the C-terminus to residue 783 (see Supplementary Figure S2C,D) and we found that it was still able to bind three zinc ions. At present, we do not understand the relevance of this additional zinc-binding domain (Zn-BD) but it is included in the construct referred to as TRIAD3A-RBR.

### The interaction between RBRs and E2 enzymes and their ubiquitin conjugates

**Figure 2:**
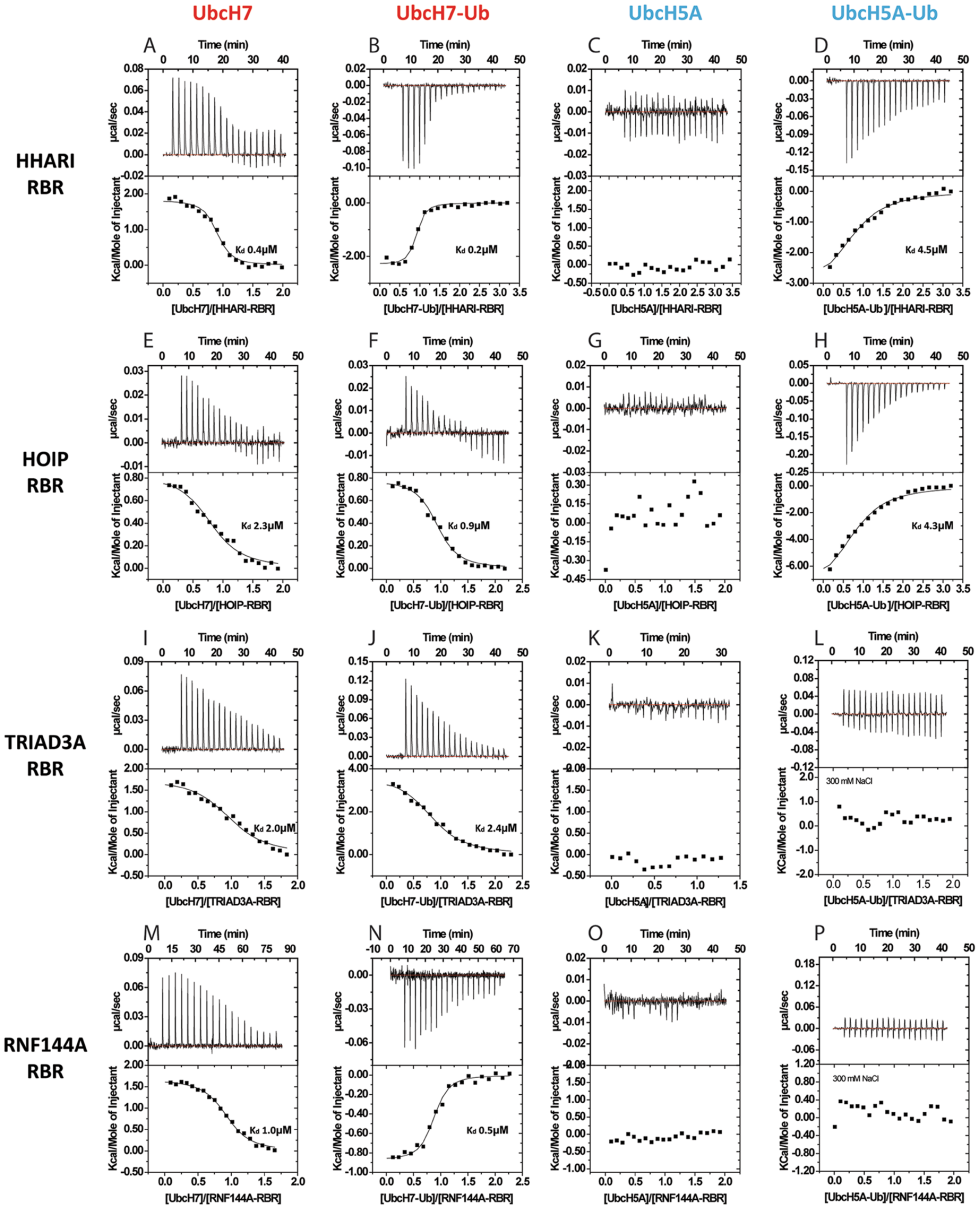
ITC analysis of the interaction of RBRs with UbcH7, UbcH5A and their ubiquitin conjugates. Isothermal titration calorimetry was used to measure the affinities of RBRs for UbcH7, UbcH5 and their ubiquitin conjugates. Experiments are reported for HHARI (**A**–**D**), HOIP (**E**–**H**), TRIAD3A (**I**–**L**) and RNF144A (**M**–**P**). Raw data (top) and normalized binding curves (bottom) are reported. Black squares indicate the normalized heat of interaction obtained per injection, while a black curve represents the best fit obtained by non-linear least-squares procedures based on a 1:1 binding model. Experiments were carried out in presence of 150 mM NaCl, apart from those reported in panels L and P, which were performed in presence of 300 mM NaCl to abolish non-specific interaction (see supplementary Figure S3).

To gain insight into the propensity of different E2 enzymes to bind RBR domains we tested their ability to bind unconjugated UbcH7 (Fig. 2A,E,I,M) and UbcH5A (Fig. 2C,G,K,O). Intriguingly, we could not detect binding of UbcH5A to any of the RBRs tested, whilst a strong interaction was observed with UbcH7. The strength of complex formation ranges between 0.4 μM and 2.3 μM, with a well-conserved thermodynamic signature: the association is largely entropy driven with a small positive enthalpy (Table 1). The observed affinity for the HHARI/UbcH7 interaction agrees well with a previous study by Duda *et al*.^10^ but is about 10-fold weaker than a recent study, which also reported an affinity of 20 μM for the HHARI/UbcH5B interaction^41^. At present it is not clear what the reason for these apparent differences is and we can only speculate that it might be linked to differences in the protein fragments used. Interestingly, conjugation of ubiquitin to UbcH7 provides only a small increase in affinity (Fig. 2B,F,J,N and Table 1) and even though the sign of the enthalpy changes for some of the RBRs tested (HHARI and RNF144A) the overall thermodynamic signatures remain conserved and binding is largely entropy driven. In contrast, UbcH5A-Ub shows a clear 1:1 interaction only with the RBR domains of HOIP and HHARI with K_d_ values around 4.5 μM, somewhat weaker than those for UbcH7-Ub. Titration of TRIAD3A and RNF144A with UbcH5A-Ub showed only a clear heat change during the first few peaks of the titration and saturation was reached after a few injections corresponding to an apparent molar ratio of E3/E2-Ub of about 0.2 (see Supplementary Figure S3A,B). To test if this sub-stoichiometric binding event might be due to a non-specific interaction we carried out titrations at increased salt concentration (300 mM NaCl instead of 150 mM). This condition indeed suppressed any apparent interaction (Fig. 2L,P and see Supplementary Figure S3C,D). Furthermore, in agreement with these ITC titrations we could not detect complex formation for TRIAD3A and RNF144A with UbcH5A-Ub during SEC-SAXS analysis (see below). Based on these observations, we conclude that under our experimental conditions TRIAD3A and RNF144A are not able to form a stable complex with UbcH5A-Ub.

**Table 1:** ITC-derived thermodynamic parameters for the association of HHARI, HOIP, TRIAD3A and RNF144A RBR and Ring1 domains with UbcH7, UbcH5A and their ubiquitin conjugates. Each experiment was repeated multiple times and the average values reported with their standard deviation.

Interactions	n	Kd (uM)	ΔH Kcal/mol	−TΔS Kcal/mol	ΔG Kcal/mol
HHARI-RBR/UbcH7	0.9 ± 0.1	0.41 ± 0.05	1.8 ± 0.2	−10 ± 0.6	−8.2 ± 0.8
HHARI-RBR/UbcH7-Ub	0.9 ± 0.1	0.20 ± 0.01	−2.3 ± 0.2	−6.6 ± 0.7	−8.9 ± 0.9
HHARI-Ring1/UbcH7	0.8 ± 0.1	0.21 ± 0.01	4.2 ± 0.5	−13.2 ± 0.4	−9.0 ± 0.9
HHARI-Ring1/UbcH7-Ub	0.8 ± 0.2	0.12 ± 0.01	3.9 ± 0.4	−13.3 ± 0.6	−9.4 ± 0.9
HHARI-RBR/UbcH5A	No binding detected				
HHARI-RBR/UbcH5A-Ub	0.8 ± 0.1	4.5 ± 0.4	−3.7 ± 0.4	−3.4 ± 0.2	−7.1 ± 0.6
HHARI-Ring1/UbcH5A-Ub	No binding detected				
HOIP-RBR/UbcH7	0.8 ± 0.2	2.3 ± 0.2	0.8 ± 0.1	−8.3 ± 0.4	−7.5 ± 0.6
HOIP-RBR/UbcH7-Ub	0.9 ± 0.1	0.9 ± 0.1	0.8 ± 0.1	−8.9 ± 0.6	−8.1 ± 0.8
HOIP-Ring1/UbcH7	1.0 ± 0.1	4.8 ± 0.5	1.4 ± 0.2	−8.5 ± 0.5	−7.1 ± 0.7
HOIP-Ring1/UbcH7-Ub	0.9 ± 0.2	4.3 ± 0.4	1.1 ± 0.2	−8.3 ± 0.5	−7.2 ± 0.7
HOIP-RBR/UbcH5A	No binding detected				
HOIP-RBR/UbcH5A-Ub	0.9 ± 0.1	4.3 ± 0.3	−8.2 ± 0.5	1.0 ± 0.1	−7.2 ± 0.7
HOIP-Ring1/UbcH5A-Ub	No binding detected				
TRIAD3A-RBR/UbcH7	1.0 ± 0.1	2.0 ± 0.1	1.8 ± 0.7	−9.4 ± 0.1	−7.6 ± 0.8
TRIAD3A-RBR/UbcH7-Ub	0.9 ± 0.1	2.4 ± 0.1	3.7 ± 0.3	−11.2 ± 0.5	−7.5 ± 0.9
TRIAD3A-Ring1/UbcH7	0.8 ± 0.2	3.2 ± 0.2	4.5 ± 0.3	−11.8 ± 0.6	−7.3 ± 0.9
TRIAD3A-Ring1/UbcH7-Ub	0.8 ± 0.2	4.3 ± 0.3	3.6 ± 0.4	−10.8 ± 0.2	−7.1 ± 0.7
TRIAD3A-RBR/UbcH5A	No binding detected				
TRIAD3A-RBR/UbcH5A-Ub	No binding detected				
TRIAD3A-Ring1/UbcH5A-Ub	No binding detected				
RNF144A-RBR/UbcH7	0.9 ± 0.1	1.0 ± 0.1	1.7 ± 0.2	−9.7 ± 0.5	−8.0 ± 0.8
RNF144A-RBR/UbcH7-Ub	0.8 ± 0.2	0.52 ± 0.04	−0.9 ± 0.1	−7.5 ± 0.7	−8.4 ± 0.9
RNF144A-Ring1/UbcH7	0.8 ± 0.2	2.2 ± 0.2	3.5 ± 0.4	−11.1 ± 0.6	−7.5 ± 0.9
RNF144A-Ring1/UbcH7-Ub	0.8 ± 0.1	1.0 ± 0.1	2.7 ± 0.4	−10.7 ± 0.5	−8.0 ± 0.8
RNF144A-RBR/UbcH5A	No binding detected				
RNF144A-RBR/UbcH5A-Ub	No binding detected				
RNF144A-Ring1/UbcH5A-Ub	No binding detected				

Taken together, these results suggest that for the interaction of UbcH5A~Ub with HHARI or HOIP, the ubiquitin moiety plays an important role in the formation of the RBR/E2~Ub complex, in agreement with the HOIP/UbcH5B-Ub structure that shows extensive contacts between ubiquitin and the RBR domain^43^. In contrast, the lack of a significant increase in affinity of RBRs for the UbcH7-Ub conjugate indicates that in this case recognition is driven primarily by contacts between RING1 and the E2. To test this model and further evaluate the role of IBR and RING2 domains in complex formation, the ITC experiments were repeated with RBR fragments containing only the RING1 domains plus the adjacent linker helices (see Supplementary Figure S4 and Table 1). These fragments are indeed able to bind to both, UbcH7 and UbcH7-Ub, with affinities similar to those observed for interaction with the entire RBR domains. On the other hand, no interaction could be detected with UbcH5A-Ub under the experimental conditions, indicating that the interaction must be weaker than ~50 µM. These data support a model where the interaction with UbcH7~Ub is largely driven by the recognition between the E2 and RING1 with only a small contribution from additional contacts of the RBR with ubiquitin. In contrast, complex formation with UbcH5A~Ub is absolutely dependent on the ability of HOIP and HHARI to contact the ubiquitin moiety conjugated to the E2.

### Relationship between RBR/E2~UB complex formation and catalytic activity

**Figure 3:**
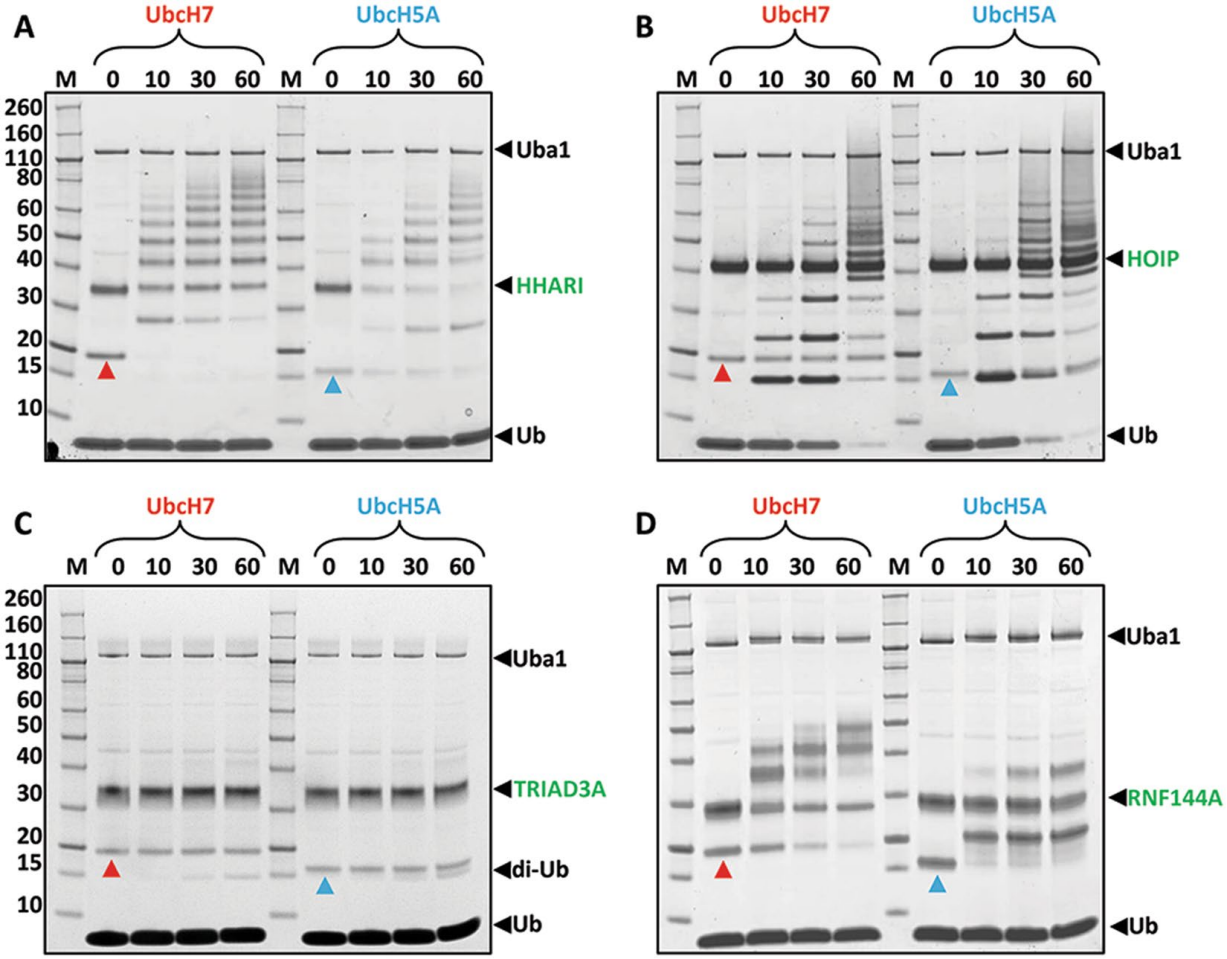
Auto-ubiquitination assays of RBRs with UbcH7 and UbcH5A. Coomassie-stained reducing SDS-gels of auto-ubiquitination assays for HHARI-RBR (**A**), HOIP-RBR (**B**), TRIAD3A-RBR (**C**) and RNF144A-RBR (**D**). The concentration of RBR used was 5 μM, whilst the concentrations of E1, E2 and Ub were 0.1, 2 and 50 μM, respectively. 10 mM ATP was added to start the reaction and samples were taken after 10, 30 and 60 minutes incubation at room temperature. Red and blue arrowheads indicate UbcH7 and UbcH5A, respectively; black arrowheads indicate E1 (Uba1), E3 and ubiquitin (Ub). In the case of TRIAD3A accumulation of di-ubiquitin (di-Ub) is observed, indicated by a black arrowhead.

Given these contrasting behaviours in recognition of UbcH5A~Ub and UbcH7~Ub conjugates by RBRs, we asked if they translate into differences in ubiquitination activity. HOIP forms free linear polyubiquitin chains in the absence of a protein substrate, whereas for the other RBRs we used auto-ubiquitination as a proxy readout for catalytic activity. It is important to note that the apparent activity seen in ubiquitination assays depends strongly on the reaction conditions used (e.g. temperature, concentrations of reactants). Since we wanted to compare the activity of four different E3s we had to optimise experimental conditions and found that the ratio between E2 and E3 concentrations is a very sensitive parameter, at least for some E3s (see Supplementary Figure S5). Our experiments showed that a good level of auto-ubiquitination could be detected across the same time range when the concentration of E3 was higher than the E2. Interestingly, the ratio between E2 and E3 does not appear to affect the activity of HOIP, but has a clear effect on that of HHARI, TRIAD3A and RNF144A. In particular the effect on HHARI is profound with long chains favoured when the assay is performed using a concentration of E2 higher than of E3; on the other hand when the assay is performed with concentrations of E2 lower than E3, or when the two enzymes have the same concentration, shorter chains are formed. On the basis of these tests we have chosen to perform the assays described in Fig. 3 with excess of E3, using the following concentrations: 0.5 μM E1, 2 µM E2, 5 µM E3 and 50 µM ubiquitin. Under these conditions, HHARI, HOIP and RNF144 showed clear ubiquitination activity with a slightly higher activity for UbcH7 with HHARI and RNF144A whereas linear chain formation by HOIP was slightly increased with UbcH5A. The results observed for HHARI and HOIP agree well with previous reports^8,10,12,41^. In contrast, no clear activity was detected for TRIAD3A. However, when the assays were repeated with a TRIAD3A construct containing a GST-tag, clear auto-ubiquitination was observed (see Supplementary Figure S6A). This difference may be due to the higher number of lysine residues available in this construct or could suggest that GST-induced dimerization increases activity. To characterise the type of ubiquitin modification formed (multi-mono or ubiquitin chain formation) ubiquitination assays were carried out with UbcH7 and lysine-less ubiquitin (Ub-K0). These experiments show that TRIAD3A and RNF144A mainly perform multi-mono ubiquitination (see Supplementary Figure S6B and D). In addition, TRIAD3A ubiquitination assays shows accumulation of some di-ubiquitin, which is mainly K63-linked, (Fig. 3C and see Supplementary Figure S6A–C). In sum, these experiments indicate that there is no clear link between the affinity of a given E2~Ub conjugate for an RBR and activity and hence that formation of a stable E3/E2~Ub complex is not required for catalytic activity. The same conclusion was recently drawn based on the analysis of HHARI^41^.

### The isolated RBR domains and their E2~Ub complexes are elongated species

Given that no structural information is currently available for an active RBR/E2-Ub complex other than HOIP^43^, we performed size exclusion chromatography coupled with SAXS analysis (SEC-SAXS) on all four RBRs in isolation and in complex with UbcH7-Ub and UbcH5A-Ub to gain novel low resolution structural insight into their conformation. The chromatographic profiles (see Supplementary Figure S7) are consistent with the assumption that the complexes of HHARI, HOIP, TRIAD3A and RNF144A with UbcH7-Ub and HHARI and HOIP with UbcH5A-Ub co-migrate as one species on the column indicating that, at the concentration used for the experiment, stable RBR/E2~Ub complexes are formed. In agreement our ITC results, no complex formation was observed for TRIAD3A and RNF144A with UbcH5A-Ub.

**Figure 4:**
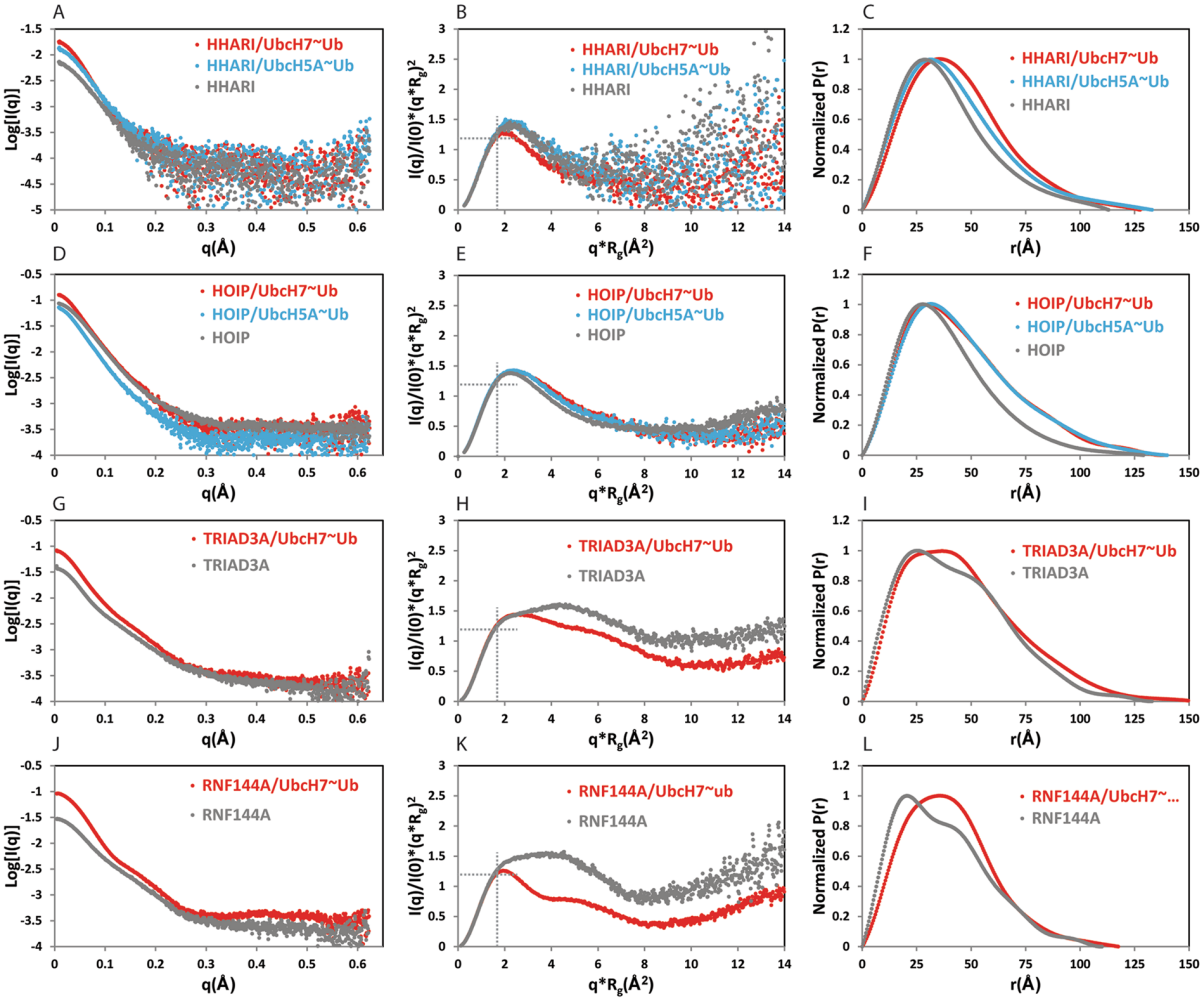
SAXS analysis of RBRs and their complexes with UbcH7~Ub and UbcH5A~Ub. Solvent-subtracted SAXS curves, Kratky plots and P(r) distributions are reported for HHARI, HHARI/UbcH7-Ub and HHARI/UbcH5A-Ub (**A**,**B**,**C**); HOIP, HOIP/UbcH7-Ub and HOIP /UbcH5A-Ub (**D**,**E**,**F**), TRIAD3A and TRIAD3A/UbcH7-Ub (**G**,**H**,**I**); RNF144A and RNF144A /UbcH7-Ub (**J**,**K**,**L**). The curves of RBRs in isolation are in grey, the curves of the complexes with UbcH7-Ub are in red and complexes with UbcH5A-Ub are in blue.

Averaged SAXS curves, corrected for the solvent effect, were obtained for all the samples by following the procedures described in materials and methods (Fig. 4A,D,G,J). The radius of gyration (R_g_) was obtained by Guinier analysis of the region of the curves at low q value and D_max_ and Volume of Porod were evaluated by the distance distribution function (P(r)) (Fig. 4C,F,I,L). The analysis of the normalized Kratky plots (Fig. 4B,E,H,K) reveals some interesting properties of the samples: in all cases the maxima of the plots are shifted from the typical values expected for globular proteins (I(q)/I(0)·(q·R_g_)^2^ = 1.104, q·R_g_ = 1.73, indicated by dashed cross lines in Fig. 4B,E,H,K to higher values indicating the presence of asymmetric particles in solution^46–48^. The asymmetry of the systems is also evident from the shape of the distance distribution function (P(r)) where distances larger than the R_g_ are extensively populated, suggesting that both, the RBRs and their complexes with E2-Ub, are elongated species. Moreover, all the samples show some degree of intramolecular flexibility, reflected by the upturn of the Krakty plots at higher q·R_g_ values. Interestingly, for TRIAD3A and RNF144A large changes in the shape of the Krakty plot and the P(r) are visible upon binding to UbcH7-Ub indicating clear changes in the overall shape of the particles and also that the intramolecular flexibility of the system is reduced. The overall conformational change, driven by E2-Ub binding, is smaller for HHARI and HOIP. The molecular weights derived from the Porod volumes and the ones obtained using the software SAXSMoW are in good agreement with molecular weights based on primary sequences indicating that the RBR domains and their complexes with UbcH7-Ub or UbcH5A-Ub are all monomeric in solution (Table 2).

**Table 2:** SAXS data-collection parameters.

*Instrument*	SEC-SAXS at SWING beamline SOLEIL											
*SEC column*	Bio-SEC. 3 (Agilent)											
*q range* (*Å* ^*−*1^)	0.0022–0.62											
*Temperature* (*°C*)	25 °C											
**Structural parameters**												
		**From ATSAS/PRIMUS**					**From ScÅtter**	**From DAMMIN**				
*Sample Name*	*Sample Concentration* (*before SEC*)	*I*(*0*) (*cm* ^*−*1^ */absorbance*) *Reciprocal Space*	*R*_*g*_ (*Å*) *Reciprocal Space*	*I*(*0*) (*cm* ^*−*1^ */absorbance*) *Real Space*	*R*_*g*_ (*Å*) *Real Space*	*D*_*max*_ (*Å*)	*Porod Volume estimate* ^a^ (*Å* ^3^)	*NSD* ^b^	*χ* ^2b^	*Mw from Porod Volume* (*V*_*Porod*_ */1*.*6*) (*Da*)	*Mw from SAXSMoW* (*kDa*)^c^	*Mw from sequence* (*kDa*)
HHARI-RBR	51 μM	0.007	31.0	0.00707 ± 0.00001	31.0 ± 0.1	112	67241 [q_max_ = 0.16]	0.63 ± 0.04	1.76	42025	42.0	35.8
HOIP-RBR	200 μM	0.088	31.4	0.08845 ± 0.00005	31.49 ± 0.05	130	66111 [q_max_ = 0.30]	0.58 ± 0.04	1.14	41320	42.5	43.4
TRIAD3A-RBR	200 μM	0.037	34.9	0.03730 ± 0.00003	34.93 ± 0.05	133	61443 [q_max_ = 0.21]	0.63 ± 0.02	2.38	38401	36.3	34.4
RNF144A-RBR	200 μM	0.029	29.7	0.02981 ± 0.00003	29.81 ± 0.05	110	44641 [q_max_ = 0.22]	0.61 ± 0.03	1.49	27900	26.6	29.0
HHARI-RBR/UbcH7~Ub	48 μM	0.018	35.1	0.01839 ± 0.00004	35.2 ± 0.1	128	115384 [q_max_ = 0.20]	0.58 ± 0.05	1.19	72115	75.3	62.5
HHARI-RBR/UbcH5A~Ub	46 μM	0.013	34.0	0.01331 ± 0.00005	34.1 ± 0.2	128	87228 [q_max_ = 0.18]	0.57 ± 0.07	2.52	54517	59.0	61.3
HOIP-RBR/UbcH7~Ub	220 μM	0.13	36.7	0.1342 ± 0.0001	36.91 ± 0.09	138	118999 [q_max_ = 0.18]	0.64 ± 0.06	0.54	74374	74.4	70.1
HOIP-RBR/UbcH5A~Ub	220 μM	0.07	36.9	0.0728 ± 0.0001	37.1 ± 0.01	140	100170 [q_max_ = 0.18]	0.58 ± 0.03	0.53	62606	74.4	68.9
TRIAD3A/UbcH7~Ub	200 μM	0.08	37.6	0.08176 ± 0.00006	37.82 ± 0.06	151	86802 [q_max_ = 0.22]	0.67 ± 0.03	2.58	54251	53.3	58.1
RNF144A-RBR/UbcH7~Ub	200 μM	0.09	31.5	0.09292 ± 0.00004	31.64 ± 0.03	118	84561 [q_max_ = 0.20]	0.50 ± 0.02	1.91	52850	54.9	55.7
**Software employed**												
*Primary data reduction*			Foxtrot									
*Data processing*			ATSAS and Scatter									
*Ab initio analysis*			DAMMIF/DAMMIN									
*Validation and averaging*			DAMAVER									
*Computation of model intensities*			CRYSOL									
*3D graphic representation*			PyMOL									

^a^The Porod Volume estimation was carried out on truncated data to the highest value of q reported in square brackets.

^b^Value derived from best DAMMIF model.

^c^ http://www.if.sc.usp.br/~saxs/.

**Figure 5:**
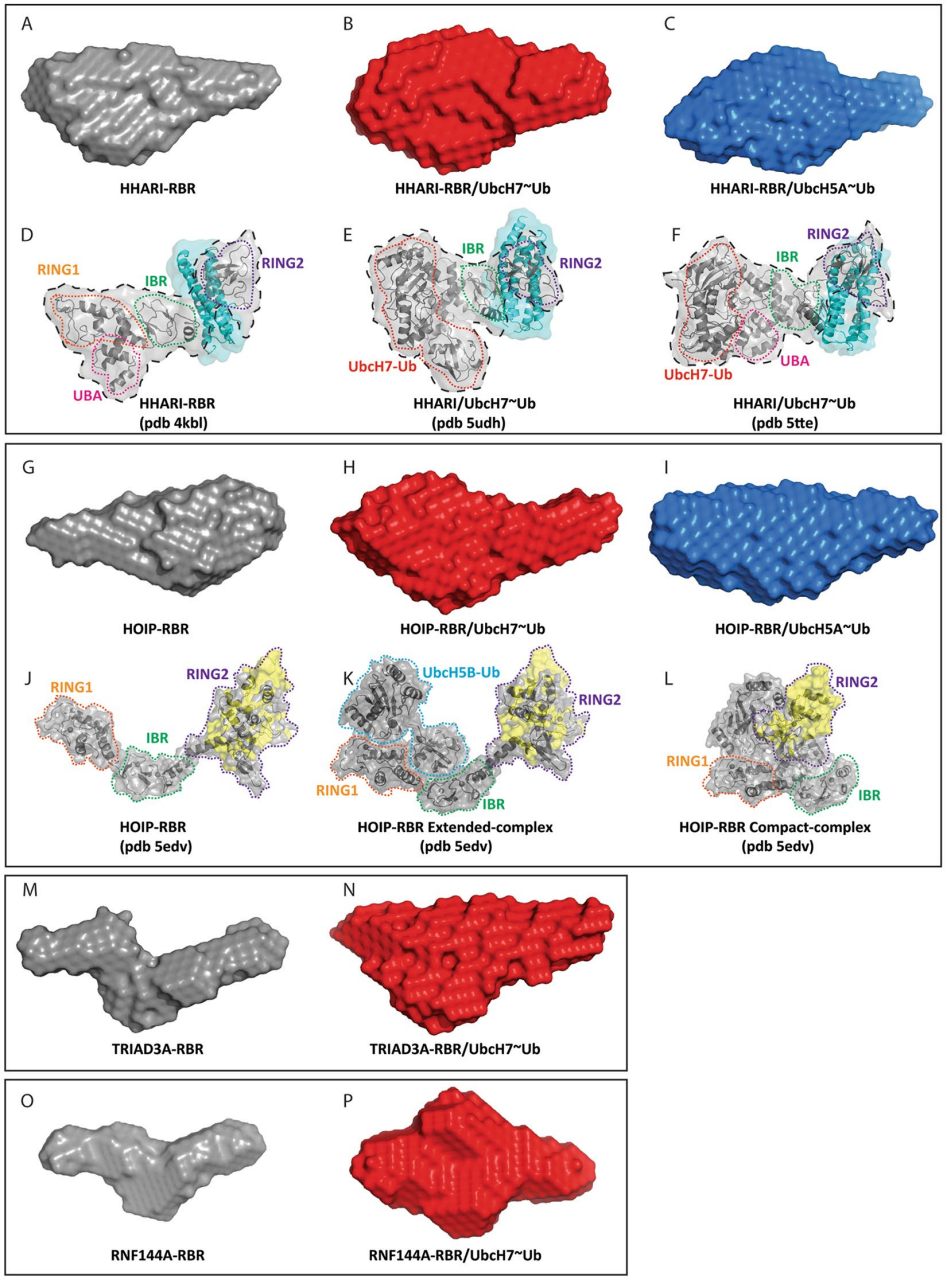
SAXS-derived envelopes and comparison with available crystal structures of HOIP/UbcH5B-Ub and HHARI/UbcH7-Ub. Low resolution SAXS-derived molecular envelopes for isolated RBRs domains (**A**,**G**,**M** and **O**) and their complexes with UbcH7-Ub and UbcH5A-Ub (**B**,**C**,**H**,**I**,**N** and **P**). Crystal structures of HHARI (PDB 4KBL) and HHARI/UbcH7-Ub (PDB 5UDH and 5TTE) are reported in panel D, E and F, respectively. The domains that constitute the RBR and E2-Ub are circled by dashed coloured lines, with UBA in pink, RING1 in orange, IBR in green, RING2 in purple and UbcH7-Ub in red. The Ariadne domain, missing in the construct used for SAXS analysis, is shown in cyan. A black dashed line contours the portions of the structures that span the same number of domains as in the construct used in our SAXS analysis. The structures of the RBR domain of HOIP, and the extended- and compact- conformations in presence of UbcH5B-Ub, derived from PDB 5EDV, are reported in (**J**–**L**). The RBR domains and the E2-Ub are circled by dashed coloured lines, with RING1 in orange, IBR in green, RING2 in purple and UbcH5B-Ub in blue. In the 5EDV structure, the RING2 of HOIP is poorly defined; therefore we have reconstructed the missing regions in yellow using the higher resolution crystal structure of RING2 reported in PDB 4LJQ.

Low-resolution envelopes were calculated for all of the samples by using the low-q region of the SAXS curve (see Table 2 for q_max_ values used in the analysis) (Fig. 5A–C,H,I,M–P). When analysing SAXS data, it is advantageous to have structural models of the molecule of interest as this allows validation of the data by back-calculation of a theoretical SAXS curve from the structural model and comparison to the experimental curve. However, this approach is only possible when the molecule in solution populates a single conformation^49^. Crystal structures are available for HOIP and HHARI, although, the HHARI construct used in our analysis does not contain the inhibitory Ariadne domain that is present in all crystal structures, while the complex of HOIP with UbcH5B-Ub lacks electron density for a proportion of the LDD domain C-terminal to RING2. Moreover, as shown by the Kratky analysis, all our samples are flexible and therefore populate in solutions a number of different conformations in solution. Hence, it is not possible at present to generate structural ensembles that fit the SAXS data as this would require the *a priori* knowledge of the flexible residues in the different constructs. Therefore only a qualitative analysis of the low resolution molecular envelopes can be performed.

The envelopes of the *apo*-RBRs (Fig. 5A,G,M,O) are all elongated and those of HHARI and HOIP show the presence of two lobes, one larger than the other. The available crystal structures of HHARI in isolation (Fig. 5D, PDB 4KBL) contain the full RBR domain together with the N-terminal UBA domain (grey surface, contoured by a black dashed line) and the auto-inhibitory Ariadne domain (cyan surface)^10^. The portion of the structure encompassing the UBA domain (Fig. 5D, circled in pink), RING1 (Fig. 5D, orange) and the IBR (Fig. 5D, green) looks strikingly similar to the large lobe of the envelope reported in Fig. 5A; therefore the smaller lobe of the HHARI-RBR envelope likely represent the location of RING2 that in the crystal structure (Fig. 5D, circled in purple) is in a different orientation probably because of the presence of the Ariadne domain. The structure of the HOIP-RBR domain (Fig. 5J) derived from the HOIP/UbcH5B-Ub complex (PDB 5EDV) is compatible with a model in which the larger lobe of the HOIP-RBR envelope (Fig. 5G) represents the portion of the molecule that contains the RING2-LLD domain (Fig. 5J, circled in purple), while the smaller lobe of the envelope represents the location of the RING1-IBR portion (Fig. 5J, circled in orange and green respectively). The molecular envelopes of isolated TRIAD3A (Fig. 5M) and RNF144A (Fig. 5O) also show an overall extended shape with three distinct regions that are reminiscent of the arrangement of the RING1, IBR and RING2 domains of one RBR polypeptide as seen in the crystal structure of the HOIP-RBR/UbcH5B-Ub complex.

In complex with UbcH7-Ub, the envelopes become larger, accounting for the presence of the E2-Ub, however, the elongated shapes are preserved in all cases (Fig. 5B,H,N,P). This set of envelopes shares similarities with the crystal structures of HHARI/UbcH7-Ub (Fig. 5E,F, PDB 5UDH and 5TTE respectively) and the extended-complex conformation observed in the HOIP/UbcH5B-Ub complex (Fig. 5K, PDB 5EDV)^41–43^. In those structures, the RBR domains are elongated with the E2s forming contacts with RING1 and the ubiquitin moieties being completely flexible (as in HHARI/UbcH7-Ub PDB 5UDH, Fig. 5E) or making some contacts with the RBR (as in HHARI/UbcH7-Ub PDB 5TTE or HOIP/UbcH5B-Ub PDB 5EDV, Fig. 5F and K respectively). An arrangement, in which the RBR is in an extended conformation and ubiquitin possess some degree of flexibility, would be in agreement with our ITC data that suggest that the binding to UbcH7-Ub is largely RING1/E2 driven with only a small effect coming from ubiquitin. The envelopes of HHARI-RBR and HOIP-RBR in complex with UbcH5A-Ub are slightly more compact than the ones obtained in complex with UbcH7-Ub but surprisingly are still elongated, suggesting that even in the presence of UbcH5A-Ub the overall shapes of the complexes are more similar to the extended-complex conformation (Fig. 5K) than to the compact-complex conformation (Fig. 5L) observed in the HOIP/UbcH5B-Ub structure.

Overall, our SAXS analysis shows that RBRs in isolation and in complex with E2~Ub, are highly dynamic species and populate mainly elongated conformations in solution. The observation that none of the available crystal structures of HHARI or for HOIP can fully recapitulate the SAXS envelopes calculated likely indicates that the available structures represent snapshots of a specific frozen conformation of complexes that otherwise possess a high intrinsic flexibility.

## Discussion

Protein ubiquitination by RBR ligases occurs via a multi-step process that involves recognition of the E2~Ub thioester intermediate by the RING1 domain, followed by ubiquitin transfer from E2 to the active site cysteine in RING2 and subsequently onto a substrate. At present, it is unclear if the nature of the E2 may influence this process and if a general mechanism of ubiquitin transfer across RBR ligases exists. To gain further insight into this reaction we characterised the interaction and activity with two E2s: UbcH5, a promiscuous E2 that is often used in biochemical and structural studies and UbcH7, which is cysteine-specific and is the physiological E2 for some, and possibly all, RBRs^8,32^.

Binding studies between four different RBRs (devoid of any auto-inhibitory domains) and the E2s in their *apo*- and ubiquitin-conjugated states showed that UbcH7 is sufficient to form a stable complex with all RBRs tested and that ubiquitin conjugation only enhances affinity to a minor degree. This observed increase is likely due to weak ubiquitin binding sites present on RBRs, such as the site on RING2 and the preceding linker recently identified by Dove and colleagues on HHARI^40^ or the binding site located on the UBA domain of HHARI reported in both structures of a HHARI/UbcH7-Ub complex (PDB 5UDH and 5TTE)^41,42^. Interestingly, two distinct orientations for the ubiquitin moiety is seen in these structures further supporting the suggestion that ubiquitin possesses a high degree of flexibility in the complex with UbcH7-Ub and is not a key anchoring point for complex formation. In contrast, stable complexes with UbcH5 could only be observed for the E2-Ub conjugate in conjunction with HHARI and HOIP and absolutely required the IBR and RING2 domains in addition to the E2-recognising RING1 domain. These observations are in good agreement with recent structural studies: the HOIP/UbcH5B-Ub complex shows an intricate network of contacts between ubiquitin and the helices connecting RING1 and IBR, the IBR domain itself, the subsequent linker and RING2, and fully supports the notion that ubiquitin is a key component in this interaction^43^. Taken together, these results suggest that RBRs can employ two different mechanisms to recognise E2~Ub conjugates: a mainly RING1/E2-driven recognition in the case of UbcH7 that appears conserved in the four different RBR ligases analysed here and a ubiquitin-driven recognition of UbcH5~Ub conjugates, at least in conjunction with HOIP and HHARI and possibly other RBRs. An exception to this observation is Parkin, which binds the ubiquitin-conjugated form of UbcH7 significantly stronger than the *apo*-form^45^. However, Parkin differs considerably from other RBR family members due to the intricate structural arrangement of its auto-inhibited form, in which the RING1 and RING2 domains are close in space and phosphorylation of the auto-inhibitory UBL domain and binding of phosphoUb are required to activate the molecule in an allosteric fashion. Different models for the structural changes accompanying Parkin activation have been put forward, including a recent model in which local conformational rearrangements may reveal novel ubiquitin-binding sites that recruit ubiquitin conjugated to the E2 and facilitate ubiquitin transfer in *trans* ^45^. Such a mechanism may be unique to Parkin and not occur in other RBRs and may explain the observed differences in E2 recognition.

The reason why RBRs have evolved two different mechanisms of recognition for UbcH7 and UbcH5 conjugates is likely based on a fundamental difference between those E2s: UbcH5 is a promiscuous and reactive E2 that can discharge ubiquitin onto lysine or cysteine residues, while UbcH7 is only cysteine reactive, despite being able to bind canonical RING domains tightly^34^. Therefore, for UbcH7~Ub, interaction with RING1 is sufficient to promote transthiolation by simply bringing the ubiquitin moiety and the catalytic cysteine of RING2 into close proximity. In contrast, for UbcH5, lysine reactivity needs to be suppressed and additional interactions are key to keep the conjugate in an open conformation that can only discharge ubiquitin onto the catalytic cysteine of RING2.

Surprisingly, given these clear differences in interaction, there is no associated pattern in terms of catalytic activity. We speculate that this might be due to a trade-off between the high affinity of UbcH7~Ub for RBRs that will ensure efficient complex formation, whereas the low affinity of UbcH5 will ensure efficient recycling. In fact, the high affinity of *apo* UbcH7 for RING1 domains seems counterproductive as the E2 would be expected to stay bound after the ubiquitin has been transferred and hence prevent the next round of ubiquitin transfer. However, it has been suggested that in cells the conjugated form of E2s is the predominant one because of efficient E1 activity^35,50,51^. Hence, it is likely that UbcH7~Ub is recycled, after discharging, by mass action displacement. On the other hand, the low affinity of UbcH5~Ub for RBRs may lower the rate of the initial encounter but once the ubiquitin is transferred the extremely low affinity of UbcH5 for RING1 will ensure that the E2 dissociates quickly after ubiquitin transfer onto RING2 and the next transfer cycle can begin; of course also in this case the mass action displacement mechanism would still hold true.

Based on our interaction studies we expected that UbcH5 and UbcH7 would promote two different architectures of the E3/E2~Ub transfer complex: an extended conformation obtained in presence of UbcH7-Ub and a more compact conformation stabilised by the extra contacts with ubiquitin when conjugated to UbcH5, as it has been observed in the HOIP/UbcH5B-Ub complex. Surprisingly, our SAXS analysis did not fully corroborate this scenario: while the complexes with UbcH7-Ub showed extended envelopes that are compatible with the absence of strong ubiquitin-RBR contacts, the envelopes of the UbcH5-Ub containing complexes looked highly similar. Nevertheless, our SAXS experiments revealed an important property of RBRs and their complexes with E2-Ub: their intrinsic flexibility. RBRs in isolation and in complex with E2~Ub are flexible species and tend to populate mainly elongated conformations. It is tempting to speculate that the two RBR conformations seen in the crystal structure of HOIP/UbcH5B-Ub, the extended and the compact conformation, could represent two possible states of the RBR/E2~Ub complex that rapidly convert into each other, with the elongated RBR conformation representing the initial E3/E2~Ub encounter complex and the closed conformation representing the ubiquitin transfer-competent intermediate, that however will be very short lived. This high level of flexibility likely explains why it has been so difficult to crystallize complexes of RBR domains in the active form, bound to the E2~Ub conjugate.

Taken together, the data presented here show that the interaction of RBRs with UbcH5 and UbcH7 occurs in different ways, likely driven by the need to supress lysine-reactivity of UbcH5 that otherwise would lead to an unproductive and potentially faulty encounter. UbcH7 has evolved to bind tightly to RING1 domains and further contacts with the conjugated ubiquitin are not required because transthiolation is the only reaction available, and this does not require activation as it is an equilibrium reaction. This may also explain why the ability of UbcH7 to form stable complexes with canonical RING domains does not need to be supressed: no catalytic cysteine is available and hence no unwanted side products can be formed. In contrast, any possible lysine reactivity of UbcH5 must be prevented to ensure that the reaction proceeds via the E3~Ub intermediate and hence extra contacts with ubiquitin ensure that no closed conformations can be formed.

All E2s, apart from UbcH7, are lysine reactive. Some may adopt closed conformations of the E2~Ub conjugate to a higher extent than UbcH5, highlighting the importance of enforcing an open E2~Ub conformation in conjunction with RBRs to ensure that the reaction proceeds via an E3~Ub intermediate to enable the ligase to transfer the ubiquitin onto the correct substrate and determine chain linkage specificity as in the case of HOIP or catalyse mono-ubiquitination as in the case of TRIAD3A and RNF144A.

## Methods

### Plasmids and protein purification

The proteins used in this work were all expressed in *E*.*coli*. The RBR domains of HOIP, HHARI and TRIAD3A were cloned in pET49b vector by using ligase-independent cloning (LIC). The RBRs were expressed as GST-fused proteins with a 3C-protease cleavage site between the tag and the protein of interest. The RBR from RNF144A was cloned into an in-house modified pET49 with an N-terminal hexa-histidine tag cleavable by 3C-protease. UbcH7 and UbcH5A in pGEX-6P1 were expressed as GST-fused proteins with a 3C-protease cleavage site. All plasmids were sequence-verified (http://www.sourcebioscience.com/). All recombinant protein samples were expressed in BL21(DE3)Gold cells at 18 °C overnight using 1 mM IPTG. Protein samples were purified following a two-step purification protocol consisting of an affinity step (using either glutathione sepharose 4B or Ni-NTA), followed by removal of the tag using 3C-protease and a size exclusion step in 25 mM Hepes pH 7.5, 150 mM NaCl, 0.5 mM TCEP. Final protein samples were concentrated and flash frozen in liquid nitrogen. UbcH5A-Ub and UbcH7-Ub were prepared using E2s with the catalytic cysteine residue mutated into lysine as previously reported^38^.

### Ubiquitination assays

Ubiquitination assays were carried out by mixing all components (E1, E2, E3 and Ub) and starting the reaction by adding ATP to a final concentration of 10 mM. Reactions were carried out at 25 °C in a final volume of 100 uL at the following concentrations, 0.5 μM E1, 2 μM E2, 5 μM E3 and 50 μM Ub (unless otherwise stated). The reaction was carried in 25 mM Hepes pH 7.5, 150 mM NaCl and samples were taken after 10, 30 and 60 minutes and quenched by adding SDS-containing buffer. Some reactions were carried out for up to 2 hours. The samples were run on reducing SDS-gels, stained with Coomassie Blue, scanned and converted to grey-scale using Adobe Photoshop.

### Isothermal titration calorimetry

ITC experiments were performed at 293 K using a Microcal iTC200 calorimeter (Malvern). The protein solutions were prepared in buffer containing 25 mM Hepes pH 7.5, 150 mM NaCl, 0.5 mM TCEP. All experiments were performed by placing the solution containing E2 or E2~Ub in the cell at concentrations between 25–30 μM and the solution containing the RBR in the syringe at concentrations between 250–300 μM. For each titration 20 injections of 2 μL were performed. Integrated data, corrected for heats of dilution, were fitted using a nonlinear least-squares algorithm to a 1:1 binding curve, using the MicroCal Origin 7.0 software package. The fitting parameters are ΔH° (reaction enthalpy change in kcal·mol-1), K_b_ (equilibrium binding constant in M^−1^), and n (number of binding sites). Each experiment was repeated at least twice and average values are reported in Table 1.

### SEC-SAXS analysis

SAXS data were collected at SOLEIL Light Source on beamline SWING. In line SEC-SAXS was performed using an Agilent 1200 HPLC system equipped with a 2.4 mL Bio-Sec. 3 (Agilent) column. Data were recorded on a PCCD170170 (AVIEX) detector and with a ~5–17 keV energy range allowing the collection of the angular range q between 0.0038–0.62 Å^−1^. Samples were loaded onto the size exclusion column previously equilibrated in 25 mM Hepes pH 7.5, 150 mM NaCl, 0.5 mM TCEP at a concentration of about 200 μM. The primary reduction of the SAXS data was performed using the software Foxtrot (http://www.synchrotron-soleil.fr/Recherche/LignesLumiere/SWING). Data processing was carried out with ATSAS (http://www.embl-hamburg.de/biosaxs/software.html) to obtain the radius of gyration (Rg), the maximum particle dimension (Dmax), the excluded particle volume (Vp) and the pair distribution function (P(r)). The program SCATTER was used to obtain the excluded particle volume (Vp). A low resolution three-dimensional *ab initio* model for all the samples was generated using the program DAMMIF, averaging the results of 25 independent runs using the program DAMAVER. CRYSOL was used to compare available structures with experimental scattering profiles. The *ab initio* models were rendered with PyMOL.

## Electronic supplementary material

Supplementary Information
