# Supplementary material for: Determinants of E2-ubiquitin conjugate recognition by RBR E3 ligases
Source: Sci Rep. 2018 Jan 8;8:68. doi: 10.1038/s41598-017-18513-5 (PMC5758712; doi:10.1038/s41598-017-18513-5)
Supplement: Supplementary file 1 — Supplementary Information [file 41598_2017_18513_MOESM1_ESM.pdf]

Determinants of E2-ubiquitin conjugate recognition by RBR E3 ligases

Luigi Martino[1], Nicholas R. Brown[1#], Laura Masino[2], Diego Esposito[1], Katrin Rittinger[1*]

[1]The Francis Crick Institute, 1 Midland Road, London NW1 1AT, UK

[2]Structural Biology Science Technology Platform, The Francis Crick Institute, 1 Midland Road, London NW1 1AT, UK

[#]Current address: School of Cellular and Molecular Medicine, University of Bristol, University Walk, Bristol, BS8 1TD. UK

[*] Corresponding author, katrin.rittinger@crick.ac.uk

Figure S1

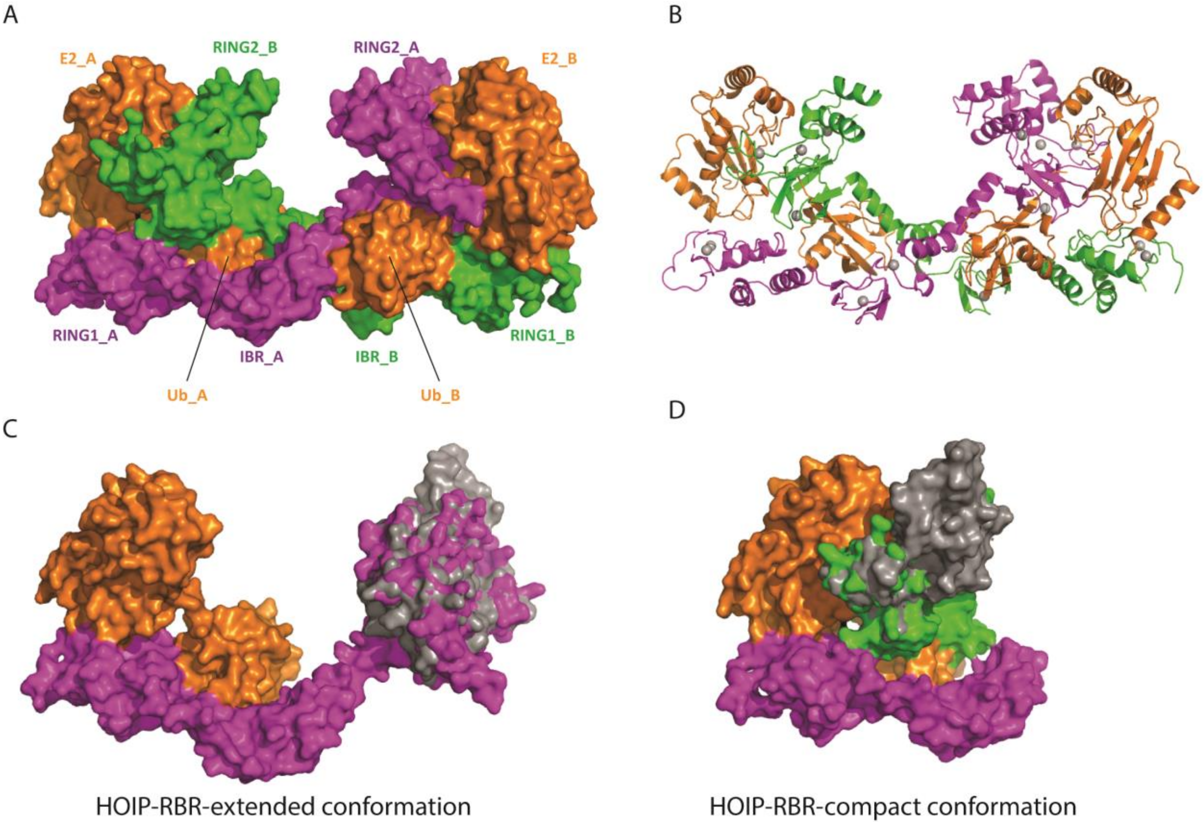

Figure S1. **Crystal structure of HOIP-RBR in complex with UbcH5B~Ub.** Crystal structure of the HOIP-RBR/UbcH5B~Ub transfer complex (PDB 5EDV) reported as surface representation (A) and as ribbon representation (B), the RING1, IBR and RING2 domain of each of two chains (purple for chain A and green for chain B) are indicated. (C) Extended-complex conformation constructed by retaining the RBR-chain A with the E2~Ub bound to its RING1 domain. (D) Compact-complex conformation assumed to be the biologically active form of the complex; E2-Ub, RING1 and IBR are from chain A whilst RING2 is from chain B. In the 5EDV structure, the RING2 of HOIP is poorly defined; therefore we have reconstructed in panels C and D the missing regions in grey using the higher resolution crystal structure of RING2 reported in PDB 4LJQ.

Figure S2

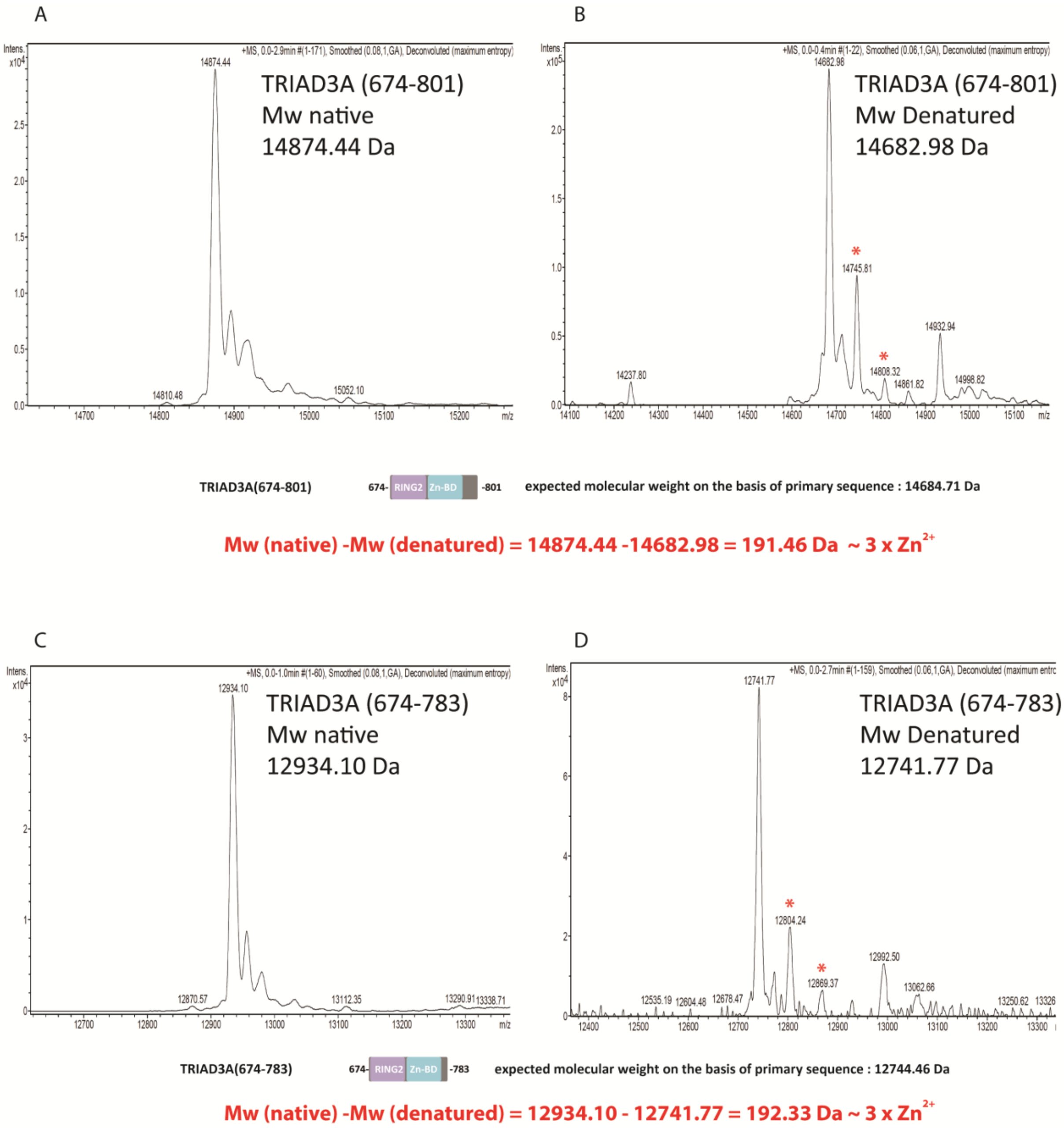

Figure S2. TRIAD3A-RBR contains a Zinc-binding region located beyond the conserved RING2 domain. Mass spectroscopy analysis of the constructs of TRIAD3A spanning residues 674-801 and 674-783 performed under native (A and C) and denaturing (B and D) conditions; the difference between the molecular weights obtained with the two methods is very close to the mass of three zinc ions (3 x 65.38 Da). In the spectra recorded under denaturing conditions satellite peaks are present at molecular weights higher than the molecular weight of the denatured protein (indicated with orange stars). They possibly correspond to portions of the samples not fully denatured and still partially bound to zinc ions, as they differ from the molecular weight of the unfolded molecules by +63 Da and +126 Da that correspond to one and two zinc ions, respectively.

Figure S3

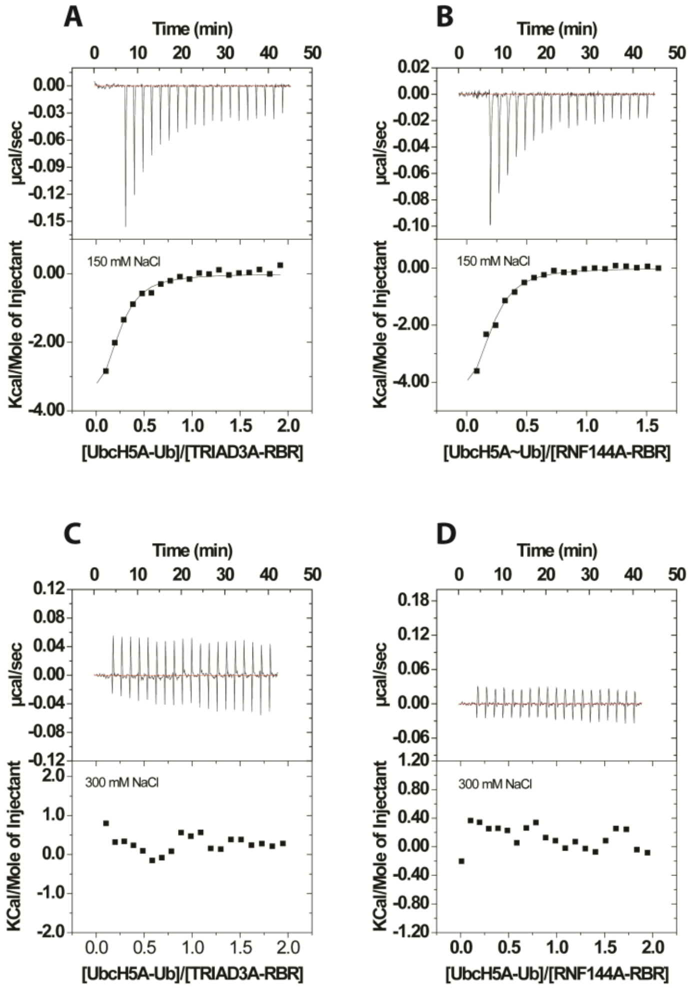

Figure S3. Non-specific interaction of UbcH5A~Ub with TRIAD3A and RNF144A. Isothermal titration calorimetry experiments reported for the interaction of TRIAD3A (A, C) and RNF144A (B, D) with UbcH5A~Ub. The experiments were carried out at two different salt concentrations, 150 and 300mM of NaCl, respectively. ITC experiments in the presence of 150mM NaCl showed unusual titration profiles with saturation occurring after a small number of injections that suggested a stoichiometry of complex formation of 0.2. This effect is lost when the experiments are repeated in a buffer containing a higher ionic strength. Therefore we conclude that the apparent non-stoichiometric interaction observed at 150mM NaCl is non-specific.

Figure S4

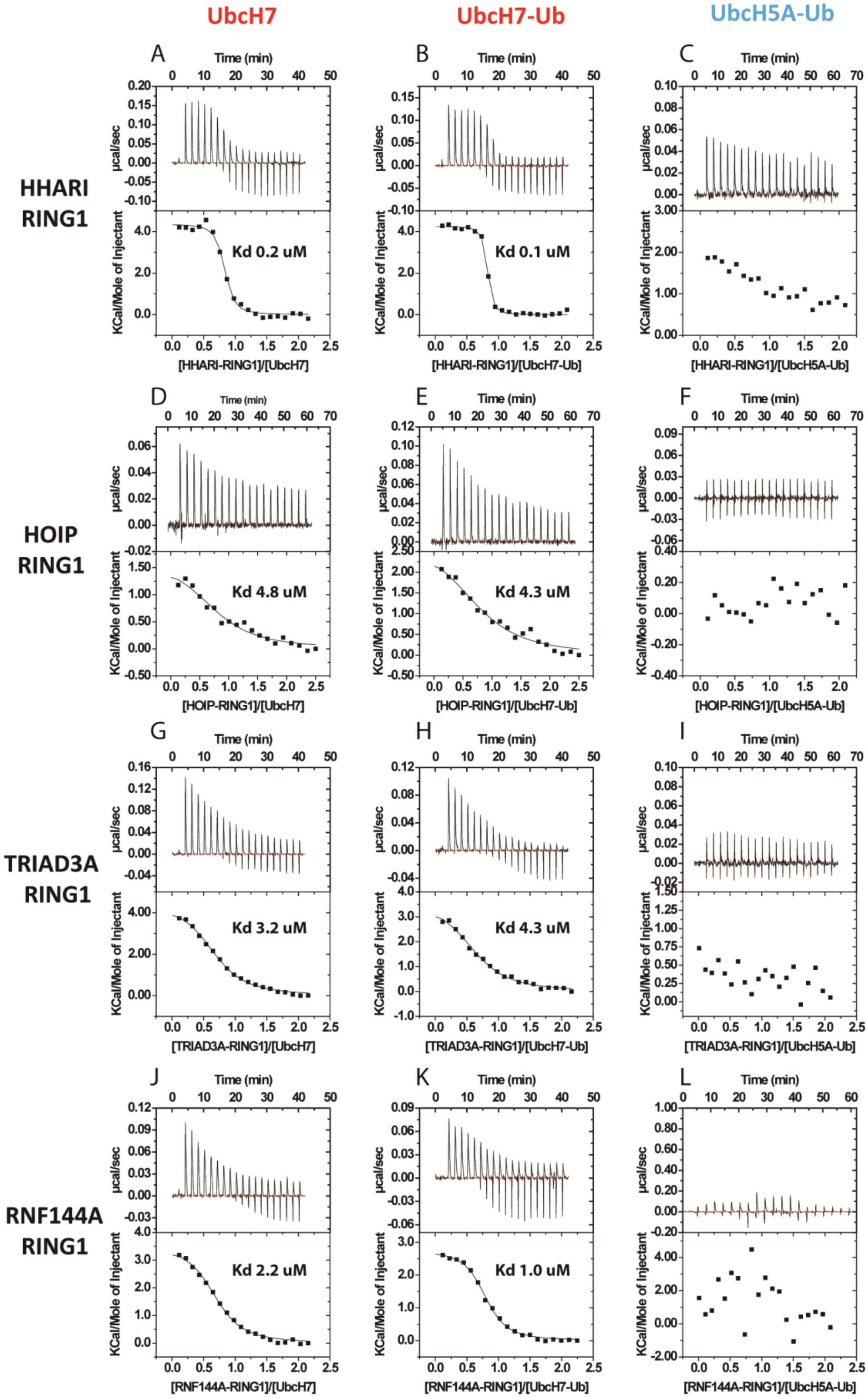
UbcH7
UbcH7-Ub
UbcH5A-Ub
HHARI RING1
HOIP RING1
TRIAD3A RING1
RNF144A RING1
A
B
C
D
E
F
G
H
I
J
K
L
Time (min)
µcal/sec
KCal/Mole of Injectant
Kd 0.2 uM
Kd 0.1 uM
Kd 4.8 uM
Kd 4.3 uM
Kd 3.2 uM
Kd 4.3 uM
Kd 2.2 uM
Kd 1.0 uM
[HHARI-RING1]/[UbcH7]
[HHARI-RING1]/[UbcH7-Ub]
[HHARI-RING1]/[UbcH5A-Ub]
[HOIP-RING1]/[UbcH7]
[HOIP-RING1]/[UbcH7-Ub]
[HOIP-RING1]/[UbcH5A-Ub]
[TRIAD3A-RING1]/[UbcH7]
[TRIAD3A-RING1]/[UbcH7-Ub]
[TRIAD3A-RING1]/[UbcH5A-Ub]
[RNF144A-RING1]/[UbcH7]
[RNF144A-RING1]/[UbcH7-Ub]
[RNF144A-RING1]/[UbcH5A-Ub]

Figure S4. ITC analysis of the interaction of RBR-RING1s with UbcH7, UbcH7-Ub and UbcH5A~Ub. Isothermal titration calorimetry was used to measure the affinities of RING1 domains for UbcH7, UbcH5 and their ubiquitin conjugates. Experiments are reported for HHARI-RING1 (A-C), HOIP-RING1 (D-F), TRIAD3A-RING1 (G-I) and RNF144A-RBR (J-L). Raw data and normalized binding curves are reported. Black squares indicate the normalized heat of interaction obtained per injection, while a black curve represents the best fit obtained by non-linear least-squares procedures based on a 1:1 binding model.

Figure S5

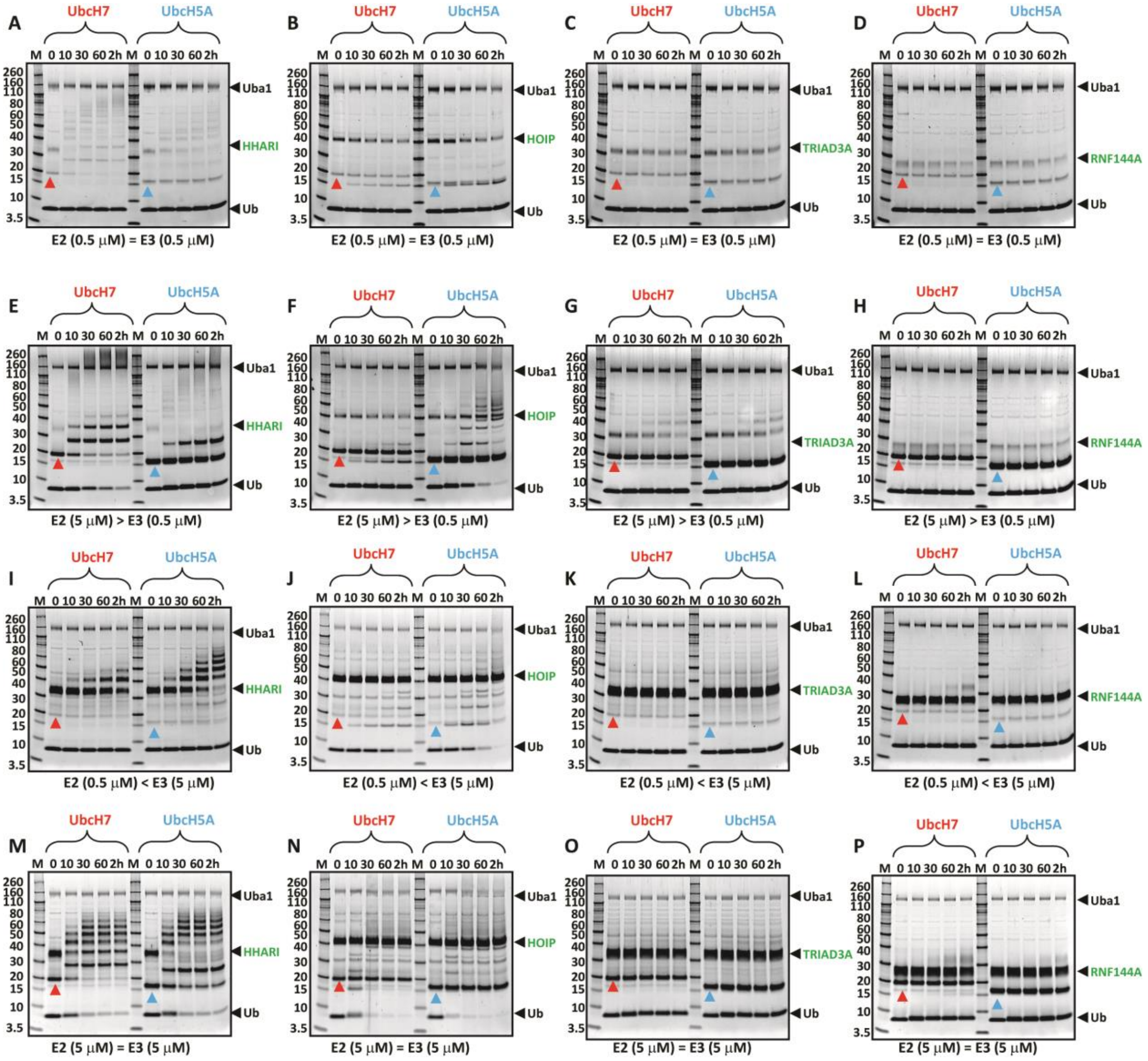

Figure S5. Assays to characterize the effect of the relative ratio between E3 and E2 on the *in vitro* auto-ubiquitination reaction. Auto-ubiquitination assays performed at 25ºC with HHARI (A, E, I, M), HOIP (B, F, J, N), TRIAD3A (C, G, K, O) and RNF144A (D, H, L, P) in the presence of UbcH7 or UbcH5A. In each experiment the concentration of E1 and ubiquitin was kept constant (0.1 μM and 10 μM, respectively) and the ratio of the concentration between the E3 and the E2 was changed. Experiments were carried out either with equal (0.5 μM or 5 μM) or different concentrations of E3 and E2. In the case of different concentrations, two conditions were investigated: a higher concentration of E3 compared to E2 (5 μM and 0.5 μM) and the opposite combination. The SDS-gels were run under reducing conditions, stained with Oriole Fluorescent Gel Stain (BioRad) and imaged using UV light excitation with a Typhoon FLA 9500 biomolecular imager (GE Healthcare). Interestingly the relative ratio between the concentration of E2 and the concentration of E3 is a sensitive parameter, at least for some E3s. We found that for HHARI, long chains are favoured when the assay is performed using a concentration of E2 higher than the concentration of E3; on the other hand when the assay is performed with a concentration of E2 lower than the concentration of E3 shorter chains are obtained. HOIP activity (free linear chain formation) does not appear to be affected by the ratio between the concentrations of E2 and E3.

TRIAD3A and RNF144A only showed low overall auto-ubiquitination activity and an opposite behaviour with respect to E3 and E2 concentrations. TRIAD3A shows increased activity when the concentration of E2 is higher than the concentration of E3. In contrast, RNF144A shows a slightly higher level of auto-ubiquitination when the concentration of E2 is lower than the E3. On the basis of these results we decided to perform auto-ubiquitination experiments (reported in Figure 3 in the main text) with a concentration of E2 lower than of E3 (2 μM and 5μM respectively). In addition, we also increased the total amount of ubiquitin and E1 to final concentrations of 50 μM and 0.5 μM because this improved the level of ubiquitination detectable.

Figure S6

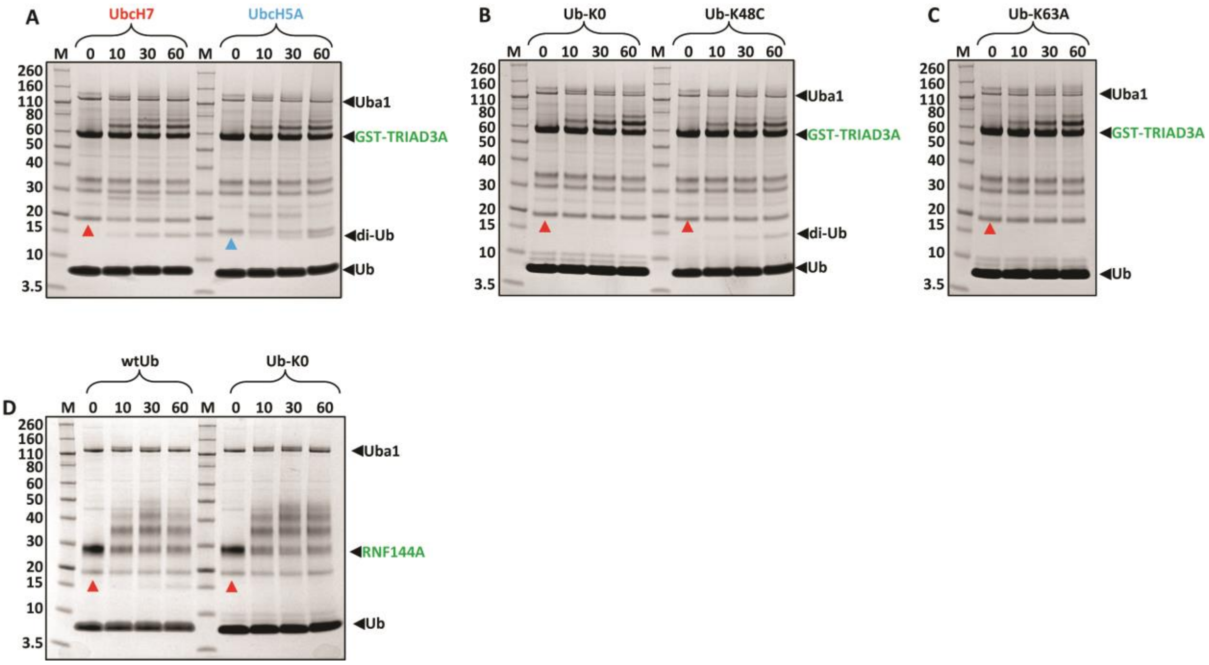

Figure S6. Auto-ubiquitination assays to characterize the ubiquitination activity of TRIAD3A and RNF144A. Auto-ubiquitination assays performed at 25 ºC with GST-TRIAD3A-RBR in the presence of UbcH7 or UbcH5A with wild type ubiquitin (A), in the presence of UbcH7 and lysine-less ubiquitin (Ub-K0) and ubiquitin-K48C (B) and with UbcH7 and ubiquitin-K63A (C). Auto-ubiquitination experiments performed with RNF144A-RBR in the presence of UbcH7 and wild type ubiquitin and lysine-less ubiquitin (D). The SDS-gels were run under reducing conditions, stained with Coomassie blue, scanned and converted to grey scale.

Figure S7

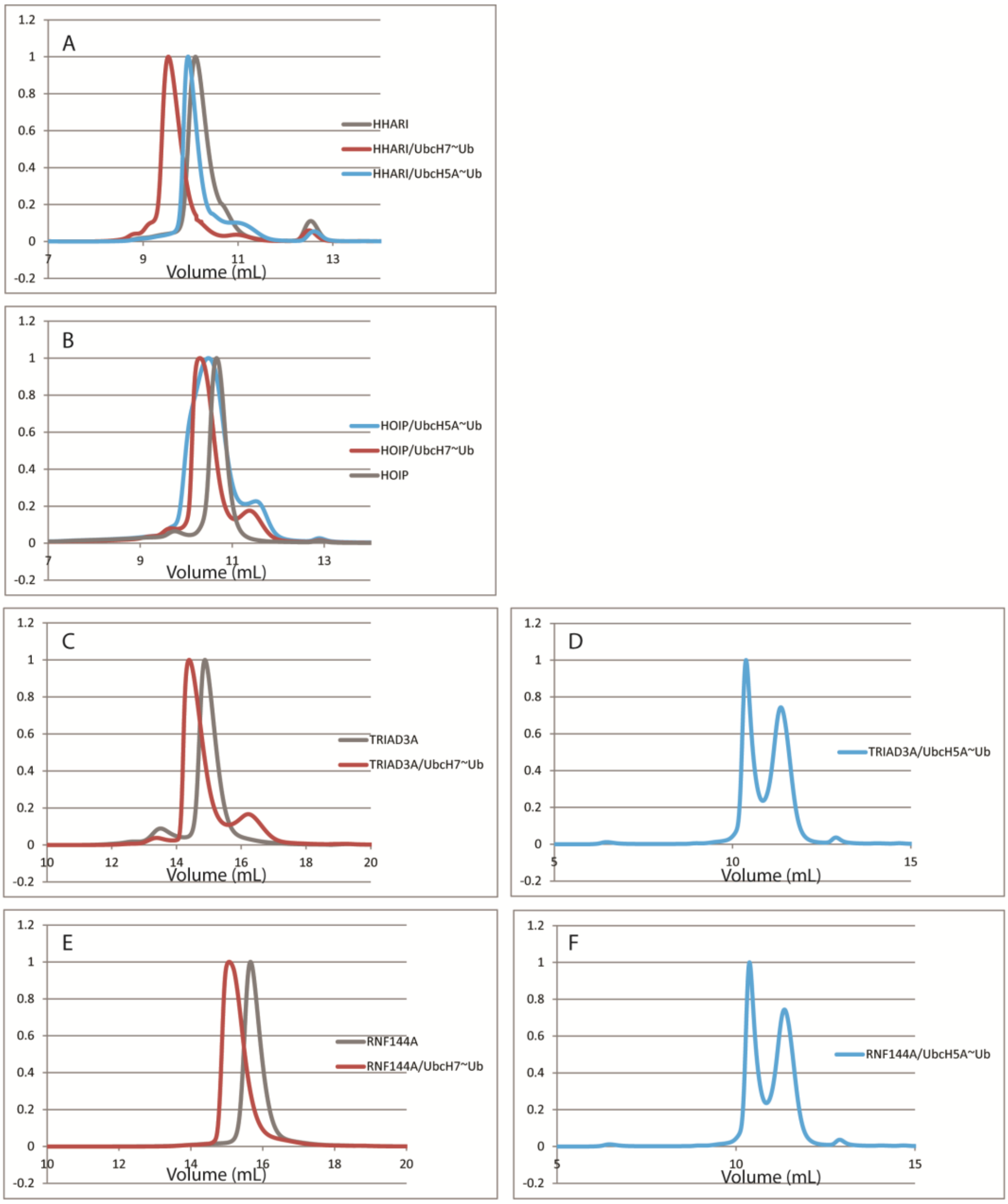

Figure S7. Size exclusion profiles of RBRs and their complexes with UbcH7~Ub and UbcH5A~Ub. Size exclusions profiles of RBRs in isolation (grey curves), in complex with UbcH7~Ub (red curves) and with UbcH5A~Ub (blue curve) for HHARI (A), HOIP (B), TRIAD3A (C-D) and RNF144A (E-F). Samples reported in the same graph were acquired at the same time with the same acquisition parameters. The RBR samples in isolation migrate with a retention volume that is smaller than the equivalent complexes with UbcH7~Ub and UbcH5A~Ub. No complex could be detected for TRIAD3A and RNF144A in the presence of UbcH5A~Ub, in those cases the RBRs and the E2~Ub migrate as separate species. SEC-SAXS experiments were acquired in two different synchrotron time slots, in particular samples reported in panel A, B, C and E were acquired in one session and samples reported in panel D and F were acquired in a second one. Slightly different experimental setups and different columns were used in the two sessions and this accounts for the different retention volumes.
